# Supplementary material for: Rehabilitation of Patients with Arthrogenic Muscular Inhibition in Pathologies of Knee Using Virtual Reality
Source: Sensors (Basel). 2023 Nov 11;23(22):9114. doi: 10.3390/s23229114 (PMC10674760; doi:10.3390/s23229114)
Supplement: Supplementary file 1 [file sensors-23-09114-s001.zip › ami_mdpi (public_access)/Asentimiento informado - Proyecto de investigación UPB - Realidad virtual AMI.pdf]

ASENTIMIENTO INFORMADO PROYECTO DE INVESTIGACIÓN: REALIDAD VIRTUAL EN EL TRATAMIENTO DE LA INHIBICIÓN MUSCULAR ARTROGÉNICA: RESPUESTA DINAMOMÉTRICA Y ELECTROMIOGRÁFICA

Investigador principal: Vera Zasulich Pérez Ariza, IEO., PhD **Celular:** 3013562840

La Universidad Pontificia Bolivariana y el Centro de Ejercicio y Fisioterapia Arthros estamos realizando un proyecto de investigación el cual tiene como **objetivo** evaluar si el uso de gafas de realidad virtual en el tratamiento de fisioterapia puede hacer que los pacientes con ciertas enfermedades en la rodilla se recuperen mejor y más rápido, por lo cual lo estamos invitando a participar.

Los **procedimientos** a los cuales usted será sometido en el Centro de Ejercicio y Fisioterapia Arthros son los siguientes:

1. Si ha sido seleccionado para las pruebas con realidad virtual, se le pondrán las gafas de realidad virtual, antes de la prueba de fuerza, actividad eléctrica muscular y los cuestionarios, para que usted vea e imite los movimientos que realizarán los personajes en el video de las gafas; sino ha sido seleccionado para las pruebas con realidad virtual, se realizarán solo los pasos 2 y 3.

2. Prueba de fuerza y actividad eléctrica muscular al mismo tiempo, con unos “parches” (electrodos de superficie) que se pondrán alrededor de la rodilla sin generar dolor, mientras extiende la rodilla durante 5s (máximo) en la silla para hacer ejercicios de pierna (extensión de rodilla). Esto, se repetirá en 3 días diferentes: en la primera sesión, en la sesión de la mitad (intermedia) y en la última sesión de fisioterapia.

3. 2 cuestionarios: uno sobre los deportes que usted hacía antes de estar enfermo de la rodilla, únicamente en la primera sesión, y otro cuestionario sobre el dolor, la dificultad que le genera moverse y hacer actividades básicas/cotidianas como caminar, subir escaleras, etc, en la primera sesión, en la sesión de la mitad y en la última.

La prueba puede generar un poco de malestar o dolor adicional a la rehabilitación. En caso de presentar mucho dolor, se suspenderá de inmediato y se realizará valoración por Fisioterapeuta; si su prueba incluye las gafas de realidad virtual puede sentir un poco de mareo.

Los **beneficios** que se obtendrán permitirán a los Profesionales Fisioterapeutas tener metodologías diversas para la atención de los pacientes y sus patologías.

Se mantendrá total **confidencialidad** de la información obtenida y suministrada por usted para esta investigación, es decir, no le diremos a nadie sus respuestas y resultados de las mediciones; solo sabrán las personas que forman parte del equipo de este estudio.

Facultad de Ingeniería – Universidad Pontificia Bolivariana Investigador principal: Vera Zasúlich Pérez Ariza
Estudiante de maestría: Juan Pablo Flórez Fonnegra
juan.florezf@upb.edu.co +57 3103552012

La participación es voluntaria, es decir, aun cuando tus Papás o adulto responsable a cargo hayan dicho que puedes participar, puedes decir que no. También es importante que sepas que, si en algún momento ya no quieres continuar en el estudio, no habrá ningún problema, o si no quieres responder alguna pregunta en particular, tampoco habrá problema. Usted está en todo su derecho de retirarse de la prueba en cualquier momento.

Si aceptas participar, por favor pon una X en el cuadro de abajo y escribe tu nombre y el de tu acompañante:

☐ SI QUIERO PARTICIPAR

Nombre
____

Nombre adulto responsable (Papá/Mamá/Hermano/Hermana/Tío/Tía/Abuelo/Abuela)
____

Investigador
Certifico que he dado la información y explicación al participante acerca del estudio y de la información contenida en el asentimiento informado de la presente investigación, respondiendo las dudas o preguntas realizadas por este y sin ejercer ninguna presión para su participación.

Firma ______________________
Nombre Vera Zasúlich Pérez Ariza
CC _______*43266979*

El presente consentimiento se firma en Medellín el ____/_______/2022 (día/mes/año).

Facultad de Ingeniería – Universidad Pontificia Bolivariana Investigador principal: Vera Zasúlich Pérez Ariza
Estudiante de maestría: Juan Pablo Flórez Fonnegra
juan.florezf@upb.edu.co +57 3103552012
