# Supplementary material for: Rehabilitation of Patients with Arthrogenic Muscular Inhibition in Pathologies of Knee Using Virtual Reality
Source: Sensors (Basel). 2023 Nov 11;23(22):9114. doi: 10.3390/s23229114 (PMC10674760; doi:10.3390/s23229114)
Supplement: Supplementary file 1 [file sensors-23-09114-s001.zip › ami_mdpi (public_access)/Consentimiento informado - Proyecto de investigación UPB - Realidad virtual AMI.pdf]

CONSENTIMIENTO INFORMADO PROYECTO DE INVESTIGACIÓN: REALIDAD VIRTUAL EN EL TRATAMIENTO DE LA INHIBICIÓN MUSCULAR ARTROGÉNICA: RESPUESTA DINAMOMÉTRICA Y ELECTROMIOGRÁFICA

Investigador principal: Vera Zasulich Pérez Ariza, IEO., PhD **Celular:** 3013562840

La UPB y el Centro de Ejercicio y Fisioterapia Arthros estamos realizando un proyecto de investigación el cual tiene como **objetivo** evaluar si la incorporación de la realidad virtual puede hacer que los pacientes con patologías de rodilla se recuperen más rápido y se adhieran más al tratamiento, por lo cual lo estamos invitando a participar.

Para esto, será necesario analizar, determinar y distribuir mediante software en dos grupos (sin/con realidad virtual) a los pacientes candidatos a la presente investigación, de manera que no interrumpa el proceso fisioterapéutico de los tratados.

Los **procedimientos** a los cuales usted será sometido en el Centro de Ejercicio y Fisioterapia Arthros están establecidos de la siguiente manera:

1. Valoración dinamométrica y electromiográfica simultánea en extensión de rodilla durante 5s (máximo) en la sesión inicial, media y fina, lo cual significa que con la ayuda de unos equipos médicos no invasivos tomaremos señales de la fuerza que realiza y la señal de sus músculos cuando usted extiende la pierna. Para ello, será necesario adherirle de forma indolora, electrodos de superficie alrededor de la rodilla.

2. 2 encuestas: un cuestionario sobre actividad deportiva antes de la lesión que se diligencia sólo una vez y otro cuestionario sobre la capacidad funcional-motora del paciente en la sesión inicial, media y final.

La prueba puede generar un poco de malestar o dolor adicional a la rehabilitación que usted hace, sin embargo, debe ser tolerable. En caso de presentar **molestias no tolerables**; ésta se suspenderá de inmediato y se realizará valoración por Fisioterapeuta al paciente. Si su prueba incluye realidad virtual puede sentir en algún momento durante o después de la prueba un poco de mareo. No hemos previsto ningún riesgo adicional.

Los **beneficios** derivados del conocimiento que se obtendrán darán herramientas e información cuantitativa a los Profesionales Fisioterapeutas para la atención de los pacientes y sus patologías.

Se mantendrá total **confidencialidad** de la información obtenida y suministrada por usted para esta investigación; ésta será tratada estadísticamente de manera grupal y sin individualizar a los involucrados en los resultados finales.

Usted está en todo su derecho de desistir de la prueba en cualquier momento.

Declaración de consentimiento informado

Facultad de Ingeniería – Universidad Pontificia Bolivariana Investigador principal: Vera Zasúlich Pérez Ariza
Estudiante de maestría: Juan Pablo Flórez Fonnegra
juan.florezf@upb.edu.co +57 3103552012

Yo ____, mayor de edad e identificado con CC. __________________________, de __________________, actuando en nombre propio o como responsable de ____.

MANIFIESTO QUE he sido informado de los propósitos de esta investigación, que he podido preguntar y aclarar las dudas y por ello **he decidido participar de manera voluntaria**. Tengo conocimiento que puedo retirar mi consentimiento cuando lo considere oportuno. Para constancia de ello firmo a continuación:

Firma ________________________________
Nombre ________________________________
CC ________________________________

Testigos
Con mi firma certifico que estuve presente durante la discusión de la información presentada en este formato de consentimiento informado, las dudas fueron resueltas y la decisión del participante es voluntaria.

Testigo 1
Firma ________________________________
Nombre ________________________________
CC ________________________________

Testigo 2
Firma ________________________________
Nombre ________________________________
CC ________________________________

Investigador
Certifico que he dado la información y explicación al participante acerca del estudio y de la información contenida en el consentimiento informado de la presente investigación, respondiendo las dudas o preguntas realizadas por este y sin ejercer ninguna presión para su participación.

Firma _____________________
Nombre Vera Zasúlich Pérez Ariza
CC *43266979*

El presente consentimiento se firma en Medellín el ____/_______/2022 (día/mes/año).

Facultad de Ingeniería – Universidad Pontificia Bolivariana Investigador principal: Vera Zasúlich Pérez Ariza
Estudiante de maestría: Juan Pablo Flórez Fonnegra
juan.florezf@upb.edu.co +57 3103552012
