# Supplementary material for: Rehabilitation of Patients with Arthrogenic Muscular Inhibition in Pathologies of Knee Using Virtual Reality
Source: Sensors (Basel). 2023 Nov 11;23(22):9114. doi: 10.3390/s23229114 (PMC10674760; doi:10.3390/s23229114)
Supplement: Supplementary file 1 [file sensors-23-09114-s001.zip › ami_mdpi (public_access)/Nivel de importancia variables desenlace.pdf]

NIVEL DE IMPORTANCIA VARIABLES DESENLACE

El cuestionario de - NIVEL DE IMPORTANCIA VARIABLES DESENLACE - lo invita a usted como Profesional Fisioterapeuta, a asignar de acuerdo a su conocimiento, experiencia y criterio, la importancia (en %) de las variables a continuación mencionadas en la evolución de los pacientes, de manera que en su ejercicio Profesional le permita decretar la recuperación de este.

Esto, con la finalidad de ponderar las variables desenlace en el proyecto de investigación - SEGUIMIENTO AL PROCESO DE REHABILITACIÓN DE PACIENTES CON INHIBICIÓN MUSCULAR ARTROGÉNICA EN PATOLOGÍAS DE RODILLA USANDO REALIDAD VIRTUAL - .

El Centro de Fisioterapia y Ejercicio Arthros en conjunto con la Universidad Pontificia Bolivariana - sede Medellín, presentan el siguiente cuestionario con la finalidad de obtener evidencia científica e investigativa sobre la metodología para el tratamiento fisioterapéutico en pacientes con patologías de rodilla. Este permitirá analizar los resultados como parte del trabajo de grado para obtener el título de Maestría en Ingeniería.

Toda la información ingresada será tratada y presentada estadísticamente; en ningún momento los datos e información serán individualizados.

Centro de Fisioterapia y Ejercicio Arthros
Universidad Pontificia Bolivariana - sede Medellín

jpff90@hotmail.com (not shared) Switch account

* Required

Nombre y apellidos: *

Your answer

Fecha *

Date

dd/mm/aaaa

POR FAVOR TENGA PRESENTE QUE TODAS LAS VARIABLES DEBEN SUMAR 100%

Cambios en el nivel de dolor *

Choose

Cambios en la rigidez muscular *

Choose

Cambios en la funcionalidad *

Choose

Cambios en la fuerza *

Choose

Cambios en la actividad eléctrica muscular *

Choose

Submit

Clear form

Never submit passwords through Google Forms.

This content is neither created nor endorsed by Google. Report Abuse - Terms of Service - Privacy Policy

Google Forms
