# Supplementary figures and images for: Rehabilitation of Patients with Arthrogenic Muscular Inhibition in Pathologies of Knee Using Virtual Reality
Source: Sensors (Basel). 2023 Nov 11;23(22):9114. doi: 10.3390/s23229114 (PMC10674760; doi:10.3390/s23229114)

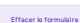

Google Forms

Supplement: Supplementary file 1 [file sensors-23-09114-s001.zip › ami_mdpi (public_access)/Cuestionario WOMAC - AMI.pdf]
