# Supplementary material for: Rehabilitation of Patients with Arthrogenic Muscular Inhibition in Pathologies of Knee Using Virtual Reality
Source: Sensors (Basel). 2023 Nov 11;23(22):9114. doi: 10.3390/s23229114 (PMC10674760; doi:10.3390/s23229114)
Supplement: Supplementary file 1 [file Cuestionario actividad deportiva - AMI.pdf › ami_mdpi (public_access)/Cuestionario actividad deportiva - AMI.pdf]

CUESTIONARIO ACTIVIDAD DEPORTIVA

El cuestionario de actividad deportiva permite determinar la calidad, intensidad y frecuencia de actividades físicas que realizaba el paciente antes de su patología; esto, con la finalidad de categorizar los niveles de actividad deportiva y comparar los tiempos de recuperación que puede tomarle al paciente de acuerdo a sus hábitos deportivos.

El Centro de Fisioterapia y Ejercicio Arthros en conjunto con la Universidad Pontificia Bolivariana - sede Medellín, presentan el siguiente cuestionario con la finalidad de obtener evidencia científica e investigativa sobre la metodología para el tratamiento fisioterapéutico en pacientes con patologías de rodilla. Éste permitirá analizar los resultados como parte del trabajo de grado para obtener el título de Maestría en Ingeniería.

Toda la información ingresada será tratada y presentada estadísticamente; en ningún momento los datos e información serán individualizados.

Centro de Fisioterapia y Ejercicio Arthros
Universidad Pontificia Bolivariana - sede Medellín

Connectez-vous à Google pour enregistrer votre progression. En savoir plus

*Obligatoire

ARTHROS
CENTRO DE FISIOTERAPIA Y EJERCICIO

A Ω UPB

Nombre y apellidos: *

Votre réponse

Fecha de nacimiento: *

Date
mm/dd/yyyy

Género *

- ○ Femenino
- ○ Masculino

Patología actual:

- ☐ Ruptura de ligamento cruzado anterior
- ☐ Artrosis
- ☐ Tendinopatía patelar

Otra ¿cuál?

Votre réponse

Extremidad afectada: *

- ○ Derecha
- ○ Izquierda
- ○ Ambas

Seleccione las actividades deportivas que realizaba en cada categoría antes de la lesión:

Votre réponse

Actividad de resistencia (aeróbica)

- ☐ Correr
- ☐ Trotar
- ☐ Nadar
- ☐ Caminar vigorosamente
- ☐ Ciclismo
- ☐ Bailar
- ☐ Saltar
- ☐ Remar
- ☐ Aeróbicos
- ☐ Montar patineta
- ☐ Tenis
- ☐ Fútbol
- ☐ Baloncesto
- ☐ Hockey
- ☐ Caminadora/máquina elíptica
- ☐ Esquí
- ☐ Patinaje
- ☐ Ninguna

Otra ¿cuál?

Votre réponse

Fortalecimiento (fuerza)

- ☐ Levantamiento de pesas
- ☐ Subir escaleras
- ☐ Flexiones de brazo
- ☐ Abdominales
- ☐ Cavar en la huerta o el jardín
- ☐ Sentadillas
- ☐ Crossfit
- ☐ Ninguna

Otra ¿cuál?

Votre réponse

Equilibrio (coordinación)

- ☐ Extensiones del cuerpo
- ☐ Caminar lentamente
- ☐ Gimnasia olímpica
- ☐ Marcha
- ☐ Estiramientos
- ☐ Ninguna

Otra ¿cuál?

Votre réponse

Estiramiento/Flexibilidad

- ☐ Yoga
- ☐ Taichi
- ☐ Pilates
- ☐ Ninguna

Otra ¿cuál?

Votre réponse

Días a la semana que hace actividad física o deporte programado, por lo menos por 45 minutos cada vez: *

- ○ 0
- ○ 1
- ○ 2
- ○ 3
- ○ 4
- ○ 5
- ○ 6
- ○ 7

Promedio de horas que entrena a la semana: *

- ○ 0
- ○ 1 - 5
- ○ 6 - 10
- ○ 11 - 15
- ○ 16 - 20
- ○ 21 - 25
- ○ 26 - 30
- ○ 31 - 35
- ○ 36 - 40

Envoyer   Effacer le formulaire

N'envoyez jamais de mots de passe via Google Forms.

Ce contenu n'est ni rédigé, ni cautionné par Google. Signaler un cas d'utilisation abusive - Conditions d'utilisation - Règles de confidentialité

Google Forms
